# An executive function subtype of PTSD with unique neural markers and clinical trajectories

**DOI:** 10.1038/s41398-022-02011-y

**Published:** 2022-06-27

**Authors:** Audreyana Jagger-Rickels, David Rothlein, Anna Stumps, Travis Clark Evans, John Bernstein, William Milberg, Regina McGlinchey, Joseph DeGutis, Michael Esterman

**Affiliations:** 1 National Center for PTSD (NCPTSD), VA Boston Healthcare System, Boston, MA USA; 2 Boston Attention and Learning Lab (BALLAB), VA Boston Healthcare System, Boston, MA USA; 3 Department of Psychiatry, Boston University School of Medicine, Boston, MA USA; 4 Department of Psychological and Brain Sciences, University of Delaware, Newark, DE USA; 5 Translational Research Center for TBI and Stress Disorders (TRACTS), VA Boston Healthcare System, Boston, MA USA; 6 Department of Psychiatry, Harvard Medical School, Boston, MA USA; 7 Geriatric Research, Education and Clinical Center (GRECC), VA Boston Healthcare System, Boston, MA USA; 8 Neuroimaging Research for Veterans (NeRVe) Center, VA Boston Healthcare System, Boston, MA USA

**Keywords:** Human behaviour, Diagnostic markers, Predictive markers

## Abstract

Previous work identified a cognitive subtype of PTSD with impaired executive function (i.e., impaired EF-PTSD subtype) and aberrant resting-state functional connectivity between frontal parietal control (FPCN) and limbic (LN) networks. To better characterize this cognitive subtype of PTSD, this study investigated (1) alterations in specific FPCN and LN subnetworks and (2) chronicity of PTSD symptoms. In a post-9/11 veteran sample (*N* = 368, 89% male), we identified EF subgroups using a standardized neuropsychological battery and a priori cutoffs for impaired, average, and above-average EF performance. Functional connectivity between two subnetworks of the FPCN and three subnetworks of the LN was assessed using resting-state fMRI (*n* = 314). PTSD chronicity over a 1–2-year period was assessed using a reliable change index (*n* = 175). The impaired EF-PTSD subtype had significantly reduced negative functional connectivity between the FPCN subnetwork involved in top-down control of emotion and two LN subnetworks involved in learning/memory and social/emotional processing. This impaired EF-PTSD subtype had relatively chronic PTSD, while those with above-average EF and PTSD displayed greater symptom reduction. Lastly, FPCN-LN subnetworks partially mediated the relationship between EF and PTSD chronicity (*n* = 121). This study reveals (1) that an impaired EF-PTSD subtype has a specific pattern of FPCN-LN subnetwork connectivity, (2) a novel above-average EF-PTSD subtype displays reduced PTSD chronicity, and (3) both cognitive and neural functioning predict PTSD chronicity. The results indicate a need to investigate how individuals with this impaired EF-PTSD subtype respond to treatment, and how they might benefit from personalized and novel approaches that target these neurocognitive systems.

## Introduction

Heterogeneity in PTSD’s symptom presentation, neurobiology, treatment efficacy, and longitudinal course has impeded progress in preventing and treating this disorder. To address this issue, researchers have begun to identify potential subtypes of PTSD, based on a range of clinical, behavioral, and biological indicators, that may help explain this heterogeneity. One approach to understanding subtypes of PTSD has been through the examination of cognition, as cognitive impairments in memory, attention, and executive functioning may underlie a number of fundamental aspects of the disorder. Recent work has suggested that cognitive subtypes of PTSD, or PTSD with specific patterns of cognitive dysfunction, have unique clinical characteristics, longitudinal clinical trajectories, and treatment efficacy [1–3].

We recently found evidence for a cognitive subtype of PTSD with impaired executive functioning. Initially, in a large sample of post-9/11 veterans, we found that PTSD was associated with reduced negative resting-state connectivity between the frontal parietal control network (FPCN) and limbic network (LN) [3]. The FPCN is thought to support executive functions (EF) like goal maintenance, cognitive flexibility, and inhibitory control, whereas the LN is important for processing and learning emotional and threatening information [4]. Aberrations in this FPCN-LN circuitry are commonly observed in PTSD and are a core feature of many neurobiological models of PTSD [4, 5] (e.g., EF and emotional regulation models [6, 7]). Critically, we identified that this connectivity marker was most prominent *specifically* in those with both PTSD and clinically significant impairments in EF. Thus, we identified an impaired EF-PTSD subtype with a specific neural signature (aberrant FPCN-LN connectivity).

Despite this discovery, the potential translational utility of this impaired EF-PTSD subtype remains unclear, including its implications in the development and longitudinal course of PTSD, as well as treatment response. However, previous work suggests that executive functioning impairments and FPCN/LN dysfunction may increase risk for developing PTSD [7–10], contribute to the maintenance of PTSD [11], and reduce treatment efficacy [12–16]. Thus, in the current study, we hypothesized that the impaired EF-PTSD subtype would have a more chronic longitudinal course of PTSD over a 1–2-year period, compared to individuals with PTSD but no EF impairment.

A limitation of the study that characterized this impaired EF-PTSD subtype was the use of a relatively coarse network parcellation. Recent literature indicates that large-scale networks such as the FPCN and LN consist of reliable and functionally distinct subnetworks that exhibit unique connectivity patterns [17–19]. For instance, several studies have identified that the FPCN subnetwork (FPCN^A^) exhibits increased connectivity with the dorsal attention network. In contrast, a second FPCN subnetwork (FPCN^B^) exhibits increased connectivity with the default mode network [20]. These subnetworks are thought to contribute differentially to executive functioning; the FPCN^A^ predominantly supports executive control of externally-focused attention, whereas the FPCN^B^ predominantly supports executive control of internally-focused attention and emotion [19]. Similarly, different limbic subnetworks may be functionally distinct [21–26]. Thus, investigating how these subnetworks differ in the EF-subtype of PTSD could help isolate the neurocognitive dysfunction in these individuals and suggest refined targets for interventions.

In the current study, we examined neurobiological and longitudinal evidence for an impaired EF subtype of PTSD. First, we examined whether this impaired EF-PTSD subtype had unique connectivity signatures between subnetworks of the FPCN and LN. Second, we examined if this impaired EF-PTSD subtype, and its corresponding neural marker(s), were predictive of the longitudinal course of PTSD symptoms. We also explored if this subtype of PTSD had differentiable comorbidities and cognitive functioning in other related domains (e.g., attention, memory, and the Gradual Onset Continuous Performance Task [gradCPT; a computer-based measure of inhibitory control]).Together, determining if this impaired EF-PTSD subtype has specific brain markers (i.e., subnetworks) and greater symptom chronicity would substantiate its clinical relevance, help explain heterogeneity in PTSD, and point toward personalized interventions.

## Methods and materials

### Participants

**Fig. 1:**
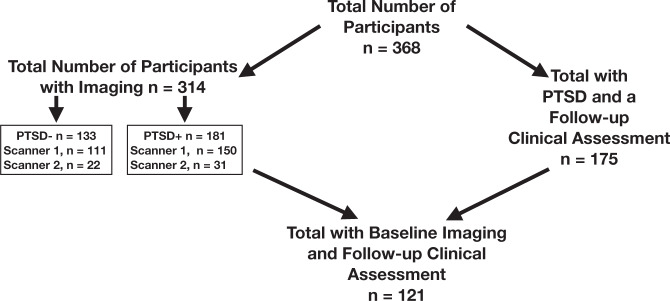
Participants. The participants available at the start of the study were collected in TRACTS between 2010 and 2017. The current study includes the first 368 participants with verified clinical data, cognitive data, who passed a performance validity test and had either neuroimaging or longitudinal clinical data. Of these 368 participants, 314 had verified resting-state fMRI and anatomical neuroimaging (181 meeting criteria for PTSD). 175 participants with PTSD had a follow-up clinical assessment, and 121 of these participants had both baseline neuroimaging and a follow-up clinical assessment. Scanner 1 was a 3 T Siemens TIM Trio scanner using a 12-channel head coil, Scanner 2 was a 3 T Siemens MAGNETOM PrismaFit scanner using a 20-channel head coil.

Participants were 368 post-9/11 veterans who served in Operation Enduring Freedom, Operation Iraqi Freedom, and Operation New Dawn that met the following criterion. All participants took part in the Translational Research Center for Traumatic Brain Injury and Stress Disorders (TRACTS) study, had verified clinical and cognitive data at baseline that passed performance validity testing (see Supplemental Methods: Assessment of PTSD, comorbidities, and demographics; Supplemental Methods: Performance Validity), participated in an MRI scan at baseline (*n* = 314), participated in a 1–2 year follow-up assessment (*n* = 175; chronicity analyses restricted those with PTSD diagnosis at baseline), or both scanning and longitudinal assessment (*n* = 121; Fig. 1). Details regarding recruitment, exclusion criterion, and assessments are described in a recent publication [27]. The sample for this study is not independent of our prior study [28], but includes an additional 97 participants. This study is thus an extension of our previous work, investigating chronicity of PTSD symptoms in those with longitudinal data, as well as subnetwork connectivity in the larger sample. All research procedures were approved by the IRB of Human Studies Research at the VA Boston Healthcare System. Participants provided informed consent and were compensated for their participation.

### Assessment of PTSD, comorbidities, and demographics

The Clinician-Administered PTSD Scale for DSM-IV (CAPS-IV [29]) was administered to diagnose PTSD and assess symptom severity. Primary analyses considered PTSD diagnosis, however, overall symptom severity and symptoms clusters, and other clinical comorbidities and demographic variables were considered in the Supplemental Results: Follow-up regression models.

### Assessment of executive functioning

EF subgroups were defined in an a priori manner using a previously validated procedure that employs a battery of six neuropsychological tests with five measures examining EF (see Table S1). Using DSM-5 criteria for mild neurocognitive impairment, impaired EF was defined as performance falling greater than one standard deviation below normative expectations on two or more neuropsychological measures of EF. In addition, we used a parallel approach to identify individuals with above-average EF as evidenced by performing at greater than one standard deviation above normative expectations on two or more neuropsychological measures [3]. All other participants scoring between these cutoff ranges were considered to have average EF. We used these previously published and normative-based cutoffs to characterize clinically significant differences in EF and increase reliability [2, 3, 30]. In addition, a continuous measure of EF was assessed with a composite mean z-score across all five measures.

### Additional cognitive measures

We examined composite measures (z-scores) of attention and verbal memory to determine the specificity of the impaired EF-PTSD subtype to EF (see Supplemental Methods: Attention and Memory Cognitive Composites; Table S1). In addition, in a subset of participants (*n* = 107), we examined performance on a computer-based cognitive assessment of sustained attention and inhibitory control known as the Gradual Onset Continuous Performance Task (gradCPT). The gradCPT is a well validated, reliable go/no-go continuous performance task [31–34]. We examined gradCPT as an independent, but mechanistically related EF measure that has been associated with PTSD is several studies [35–38]. The gradCPT has two primary measures of task ability [34]: accuracy and reaction time variability (see Supplemental Methods: gradCPT).

### Neuroimaging methods

#### Acquisition and preprocessing

Anatomical and 12 min of resting-state fMRI were acquired with a 3 T Siemens TIM Trio scanner, using a 12-channel head coil (*n* = 261) or a 3 T Siemens MAGNETOM PrismaFit scanner using a 20-channel head coil (*n* = 53). See Fig. 1 and the Supplemental Methods: MRI Acquisition for more details on scanning parameters. Scanner differences were considered as covariates in neuroimaging analyses. Preprocessing protocols matched our prior publication [3]. Details regarding preprocessing and quality control are found in the Supplemental Methods: Image Processing.

#### Brain parcellation and functional connectivity

**Fig. 2:**
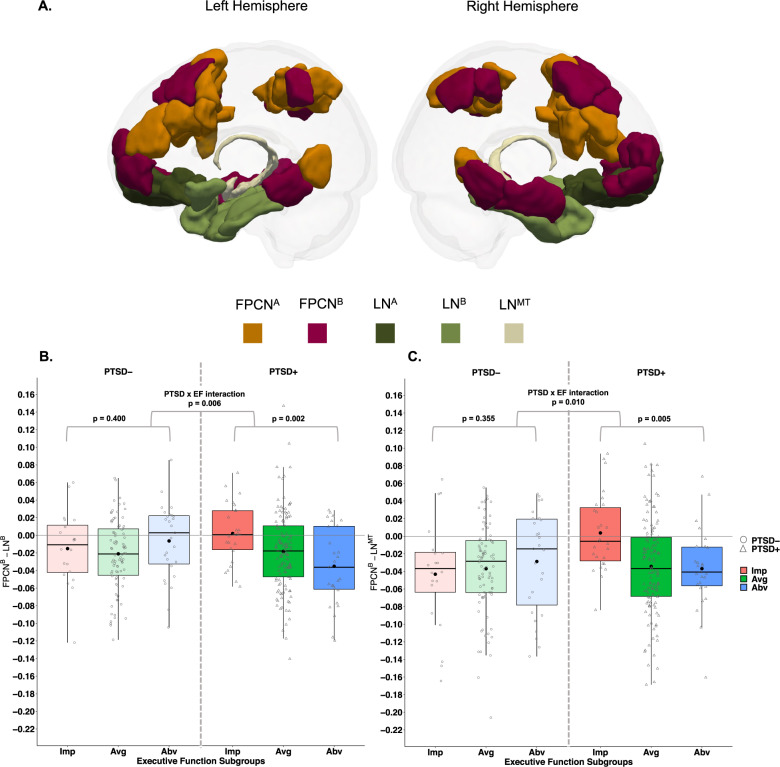
Functional connectivity across executive function subgroups. **A** Visualization of the two FPCN subnetworks (FPCN^A^, FPCN^B^), two LN (LN^A^, LN^B^) subnetworks, and the third medial temporal LN (LN^MT^). **B** PTSD and EF subgroups interacted to explain LN -FPCN connectivity, such that EF subgroups differences were present in those with PTSD. **C** PTSD and EF interacted to explain LN -FPCN connectivity, such that EF subgroup differences were present in those with PTSD. Within each box, the large dot denotes the mean, and the horizontal line denotes the median. The box indicates the interquartile range (the 25th to the 75th percentile) and vertical line from each box indicates the largest and the smallest value that fall within 1.5 times the interquartile range. EF executive function, PTSD posttraumatic stress disorder, PTSD− individuals without a PTSD diagnosis, PTSD+ individuals with a PTSD diagnosis, Imp impaired EF, Avg average EF, Abv above-average EF, LN limbic network, FPCN frontal parietal control network.

In the current study, we used a standardized cortical 17 network parcellation [39] with 200 regions of interest (or parcels). Based on our previous study [3], the current study focused on the connectivity between the FPCN and LN by selecting the two FPCN subnetworks (FPCN^A^ and FPCN^B^) and two LN subnetworks (LN^A^, LN^B^) from this cortical atlas. An additional network (LN-medial temporal; LN^MT^) included four medial temporal brain regions (bilateral amygdala and hippocampus) [40], used in our previous studies [2, 3, 41], as these structures are commonly implicated in neurobiological models of PTSD (Fig. 2A). Following preprocessing (Supplemental Methods: Brain Parcellation), average time series were extracted from each network (averaged across voxels in each parcel and then parcels within each network) and correlated (Pearson) with their respective between-network pairs (e.g., LN^A^ correlated with FPCN^A^) for a total of six between-network correlations. The connectivity (correlation) values were Fisher transformed prior to running group-level statistics.

### Chronicity of PTSD symptoms

Follow-up clinical assessments were conducted 10–90 months (mean = 25.75 months) following baseline assessments (see Participants; Fig. 1). Of these, we focused the following analyses on the 175 with a PTSD diagnosis at baseline, in order to assess chronicity of PTSD. With this additional assessment, we calculated a reliable change index to measure the chronicity of PTSD symptoms. The reliable change index is a continuous regression-based change measure developed by McSweeny et al. [42], and subsequently updated by Hilton-Bayer [43]. This method, unlike a simple subtraction-based measure, adjusts for regression to the mean, test-retest reliability, and inequality of variance. First, the total CAPS score at follow-up was adjusted for the total CAPS at baseline (residual change), while also adjusting for test-retest reliability of CAPS-IV [44] (see Supplemental Methods: Reliable Change Index). Since there was a wide range of time between baseline and follow-up assessments, we included time between baseline and follow-up as a covariate in the reliable change index analysis. This analysis controlled for any systematic differences of time between baseline and follow-up on chronicity of PTSD symptoms (e.g., those with more chronic PTSD could have shorter time between baseline and follow-up). In a subset of these participants (*n* = 91), we were able to examine medical records between baseline and follow-up to identify those who had sought treatment for PTSD. Treatment was investigated as a potential covariate in reliable change index analyses (see Supplemental Methods: Treatment).

### Statistical analysis

#### Clinical, cognitive, demographic differences between EF subgroups

Using the full sample (*n* = 368, Fig. 1), we examined if EF subgroups (impaired, average, and above average) differed in clinical symptoms, cognitive measures (in addition to EF), and demographics. To do this, we conducted linear regressions treating EF subgroups ordinally, predicting: age, gender identity, education, verbal ability, CAPS symptom clusters, mild TBI, alcohol use, anxiety, depression, sleep, pain, attention, and memory. In subsequent EF subgroup analyses (fMRI, chronicity), we considered factors that significantly differed across EF subgroups as potential covariates, to isolate the unique predictive power of EF (above and beyond these additional clinical, cognitive, and demographic correlates of EF), as well as other relevant confounds that could be related to functional connectivity (i.e., scanner, head motion) or reliable change (i.e., time between baseline and follow-up, treatment-seeking). These covariates were considered in separate analyses by isolating the effect of EF by category of covariates (e.g., demographics, clinical measures, or cognition). See Supplemental Results: Follow-up Regression Models and Table S2.

#### Subnetwork connectivity differences between EF subgroups of PTSD

We used six multiple linear regression models to determine if the impaired EF-PTSD subtype had a unique connectivity profile in one or more of the FPCN-LN subnetwork connections (*n* = 314, see Fig. 1). A significant interaction between PTSD diagnosis and EF subgroups (treated ordinally) predicting connectivity between FPCN (A and B) and LN (A, B, and MT) subnetworks would indicate that the combination of EF and PTSD, above and beyond the main effects of each, uniquely predicts subnetwork connectivity. A significant interaction would extend our previous work which found that PTSD alongside EF impairments was associated with whole-network FPCN-LN dysconnectivity in a smaller sample using a more course (7 networks) brain parcellation. The six interactions were investigated with and without specific categories of covariates (e.g., demographics, clinical, cognitive, scanner, head motion, and treatment [See Supplemental Results: Follow-up Regression Models and Table S2]). We followed up on significant interactions by investigating the relationship between EF and connectivity in those with and without PTSD, separately.

#### PTSD chronicity differences across EF subgroups of PTSD

We used linear regression to test whether EF subgroups (treated ordinally) predicted chronicity of PTSD symptoms (i.e., RCI), in those with a PTSD diagnosis at baseline (*n* = 175, see Fig. 1). This main effect was tested with and without covariates. Exploratory analyses considered whether the inclusion of FPCN-LN subnetwork connectivity improved prediction of the reliable change index and potentially mediated the effect of EF on PTSD chronicity (*n* = 121, see Fig. 1 for sample information). We conducted a mediation analysis using the Preacher and Hayes Method [45] and the Mediation package in R. First, we assessed if EF remained a significant predictor of chronicity in this smaller sample (*n* = 121), followed by determining if functional connectivity predicted chronicity while controlling for EF. Lastly, we calculated the indirect effect connectivity had on chronicity while calculating confidence intervals using a bootstrapping procedure [45].

## Results

### Clinical, cognitive, demographic differences between EF subgroups

EF subgroups differed in verbal abilities, total CAPS score (especially hyperarousal symptoms), alcohol use, sleep dysfunction, attention, and memory (*p* values < 0.038, see Table 1). Therefore, in our subsequent analyses examining whether the impaired EF-PTSD subtype had unique subnetwork connectivity, we considered these significant demographic, clinical, and cognitive measures as separate categories of covariates to ensure these brain signatures were unique to PTSD and EF (See Supplemental Results: Follow-up Regression Models, Table S2). These covariates were considered in our chronicity analysis (except for baseline PTSD symptoms which were already used to residualize the reliable change index [RCI], see Methods), determining if prediction of PTSD-chronicity was unique to EF and not driven by these related factors. We did not explicitly test for proportional differences in EF subgroups related to race. This is because there were proportionally few American Indian, Asian, Black, and Pacific Islander participants relative to White participants, limiting the statistical power to detect meaningful differences. However, the racial distribution across the subgroups are reported in Table 1.

**Table 1:** Sample characteristics.

		Total Sample (*N* = 368)				
		Imp	Avg	Abv		
		*n* = 55	*n* = 254	*n* = 59		
Demographics		Mean (SD)	Mean (SD)	Mean (SD)	*β*	*p* value
	Age	32.53 (7.48)	32.00 (8.68)	31.08 (7.37)	−0.05	0.351
	Gender (% male)	80%	89.76%	93.22%	5.70^a^	0.058
	Education	13.93 (1.69)	13.98 (1.97)	14.46 (2.02)	0.08	0.137
	Verbal Ability	99.31 (11.87)	103.87 (10.87)	110.19 (9.23)	**0.27**	**<0.001**
	Race^b^					
	American Indian	0.00%	0.00%	1.69%		
	Asian	1.82%	3.15%	1.69%		
	Black	16.36%	7.48%	5.08%		
	Pacific Islander	3.64%	0.00%	0.00%		
	White	58.18%	74.41%	94.92%		
	Medications					
	Antidepressant	27.27%	26.80%	24.56%	0.14^a^	0.933
	Antiepileptic	5.45%	2.00%	3.51%	2.14^a^	0.343
	Sedative/Hypnotic	5.45%	8.80%	7.02%	0.775^a^	0.677
	Pain	34.55%	26.00%	36.84%	3.62^a^	0.164
**Clinical**						
	CAPS-IV Total	55.27 (29.60)	51.60 (28.58)	44.17 (28.15)	**−0.11**	**0.038**
	Re-experiencing	14.05 (9.68)	13.38 (9.58)	12.03 (9.30)	−0.06	0.255
	Avoidance/Numbing	21.25 (13.20)	19.31 (13.00)	16.59 (12.41)	−0.1	0.054
	Hyperarousal	19.96 (9.94)	18.91 (9.34)	15.54 (9.11)	**−0.13**	**0.011**
	PTSD Diagnosis	63.64%	66.14%	54.24%	2.94^a^	0.230
	Mild TBI	45.45%	43.70%	45.76%	0.12^a^	0.942
	Alcohol Use	7.58 (4.86)	6.01 (3.57)	5.76 (4.40)	**−0.13**	**0.015**
	Anxiety	8.94 (8.43)	6.68 (7.44)	6.46 (8.03)	−0.09	0.098
	Depression	9.70 (9.34)	9.08 (9.77)	8.42 (9.93)	−0.04	0.491
	Sleep Dysfunction	11.04 (4.57)	9.85 (4.74)	8.85 (4.55)	**−0.13**	**0.016**
	Chronic Pain	32.29 (22.91)	30.83 (25.12)	26.68 (24.81)	−0.07	0.236
**Cognitive**						
	Attention	−0.35 (0.49)	0.05 (0.56)	0.43 (0.51)	**0.37**	**<0.001**
	Memory	−0.64 (0.85)	−0.29 (1.01)	−0.01 (0.85)	**0.18**	**<0.001**

*β* and *p* values are from regression analyses in which EF groups predicted demographics, clinical, and cognitive measures. Significant effects are bolded, and these were included as covariates in follow-up functional connectivity and chronicity analyses. Re-experiencing, avoidance/numbing, and hyperarousal scales are symptom clusters from the CAPS-IV.

*EF* executive functioning, *Imp* impaired EF, *Avg* average EF, *Abv* above-average EF, *PTSD+* Individuals with a PTSD diagnosis, *PTSD−* Individuals without a PTSD diagnosis, *Verbal Ability* total score from the Wechsler Test of Adult Reading, *CAPS-IV Total* total score from the Clinical-Administered PTSD Scale for DSM-IV, *Mild TBI* Mild military TBI flag from the Boston Assessment of Traumatic brain injury-lifetime, *Alcohol Use* average number of drinks on a drinking day from the Lifetime Drinking History, *Depression and Anxiety* total scores from Depression Anxiety Stress Scale, *Sleep Dysfunction* global sleep score from the Pittsburgh Sleep Quality Index global sleep score, *Chronic Pain* average pain in the last month score from the McGill Short Form.

^a^χ2 test was used to test for proportional differences across EF groups.

^b^No statistical test conducted due to the small size for participants that identified as American Indian, Asian, Black, and Pacific Islander.

### gradCPT differences between EF subgroups

We also investigated the relationship between EF subgroups and performance on the gradCPT, a computer-based measure of sustained attention and inhibitory control, to externally corroborate the EF measure. Both primary measures from the gradCPT (accuracy and reaction time variability) were significantly predicted by EF subgroups, indicating clinically significant differences in EF based on neuropsychological measures are also associated with these related cognitive processes (*p* values < 0.01; see Supplemental Results: gradCPT). This substantiates our EF groups using an independent measure of EF (i.e., inhibitory control).

### Subnetwork connectivity differences between EF subgroups of PTSD

We determined if the impaired EF-PTSD subtype had a unique FPCN-LN connectivity profile using six multiple linear regression models examining the main effects and interaction between PTSD diagnosis and EF subgroups predicting connectivity between FPCN (A, and B) and LN (A, B, and MT) subnetworks. We observed significant PTSD by EF interactions for FPCN^B^-LN^B^ connectivity (*β* = −0.59, *p* = 0.006, *FDR-q* = 0.018) and FPCN^B^-LN^MT^ connectivity (*β* = −0.55, *p* = 0.010; *FDR-q* = 0.021, see Table 2 and Fig. 2B, C), but not for the other FPCN-LN subnetworks (*p* values > 0.520; see Table 2). For both the FPCN^B^-LN^B^ and FPCN^B^-LN^MT^ connectivity, the significant interaction indicated reductions in negative connectivity with more impaired EF, but only for those with PTSD. These interactions remained significant after controlling for demographic, clinical, and cognitive covariate categories identified in the previous analysis (*p* values < 0.05; see Table S2) and differences in scanner parameters and head motion (*p* values < 0.012 see Table S2). These interactions also remained significant across various network parcellation sizes (*p* values < 0.016; 300–1000 parcels; see Supplemental Results and Table S3). We also investigated the interaction between EF and CAPS symptom clusters to determine if this effect was specific to re-experiencing, avoidance, or hyperarousal. This analysis revealed no evidence for specificity to any one symptom cluster (see Supplemental Results and Table S4). In sum, the PTSD by EF interaction uniquely predicted FPCN^B^-LN^B^ and FPCN^B^-LN^MT^ connectivity when accounting for a number of covariates including, demographic, clinical, cognitive correlates of EF, or differences in MRI scanners, regardless of parcellation size (Supplemental Results: Follow-up Regression Models, Table S2).

**Table 2:** Subnetwork connectivity differences between EF subgroups of PTSD.

Model			*β*		
	*Adj R*^2^	*p*	PTSD	EF Subgroups	Interaction (PTSD x EF)
FPCN^A^-LN^A^	−0.010	0.966	0.07	0.04	−0.08
FPCN^A^-LN^B^	−0.005	0.724	−0.05	−0.07	0.08
FPCN^A^-LN^MT^	0.004	0.241	0.24	0.07	−0.14
FPCN^B^-LN^A^	−0.006	0.787	0.14	0.08	−0.12
FPCN^B^-LN^B^	0.024	0.015	0.54^b^	0.07	−0.59^b^
FPCN^B^-LN^MT^	0.022	0.018	0.58^b^	0.08	−0.55^a^

Regression models were conducted (*n* = 314) to determine if PTSD diagnosis, EF subgroups, and their interaction explained FPCN-LN subnetwork connectivity.

*Adj* Adjusted, *FPCN* frontal parietal control network, *LN* limbic network, *MT* medial temporal, *EF* executive function.

^a^Indicates *p* < 0.05.

^b^Indicates *p* < 0.01.

Given these interactions, we next examined how EF subgroups predicted FPCN^B^-LN^B^ and FPCN^B^-LN^MT^ separately for those with a diagnosis of PTSD and without a diagnosis of PTSD (Fig. 2B, C). EF subgroups predicted connectivity, but only for those with PTSD (FPCN^B^-LN^B^: *Adj R*^*2*^ = 0.05, EF-*β* = −0.23, *p* = 0.002; FPCN^B^-LN^MT^: *Adj R*^*2*^ = 0.04, EF-*β* = −0.21, *p* = 0.005) and not for those without PTSD (FPCN^B^-LN^B^: *Adj R*^*2*^ = 0.002, EF-*β* = 0.08, *p* = 0.400; FPCN^B^-LN^MT^: *Adj R*^*2*^ = 0.001, EF-*β* = 0.08, *p* = 0.355). Further, using independent samples *t*-tests, we investigated differences in FPCN^B^-LN^B^ and FPCN^B^-LN^MT^ connectivity between EF subgroups in the PTSD sample (Table S5). The impaired EF-PTSD subtype had reduced negative connectivity relative to those with PTSD and average EF(FPCN^B^-LN^B^—*t*(48.60) = 2.67, *p* = 0.010; FPCN^B^-LN^MT^—*t(*45.46) = 3.92, *p* < 0.001) or above-average EF (FPCN^B^-LN^B^—*t*(48.34) = 3.49, *p* = 0.001; FPCN^B^-LN^MT^—*t*(51.95) = 3.37, *p* = 0.001). Next, we investigated if the average negative connectivity of each EF-PTSD subgroup was significantly different from zero (one-sample *t*-test). This analysis would determine if the connectivity for each EF-PTSD group was significantly negative (i.e., negative connectivity), or not (i.e., reduced negative connectivity). The connectivity for both average and above-average EF groups was significantly different from zero (average EF: FPCN^B^ ~ LN^B^ *t*(126) = −4.54, *p* < 0.001, FPCN^B^ ~ LN^MT^ *t*(126) = −7.03, *p* < 0.001; above-average EF: FPCN^B^ ~ LN^B^ *t*(26) = −4.12, *p* < 0.001, FPCN^B^ ~ LN^MT^ *t*(26) = −4.26, *p* < 0.001), whereas the connectivity for the impaired EF group was not (FPCN^B^ ~ LN^B^: *t*(24) = 0.32, *p* = 0.751; FPCN^B^ ~ LN^MT^ *t*(24) = 0.44, *p* = 0.664). These analyses indicate that the impaired EF-PTSD subtype had a unique neural signature of reduced negative connectivity between FPCN^B^-LN^B^ and FPCN^B^-LN^MT^ compared to the average and above-average EF-PTSD groups.

### PTSD chronicity differences across EF subgroups

**Fig. 3:**
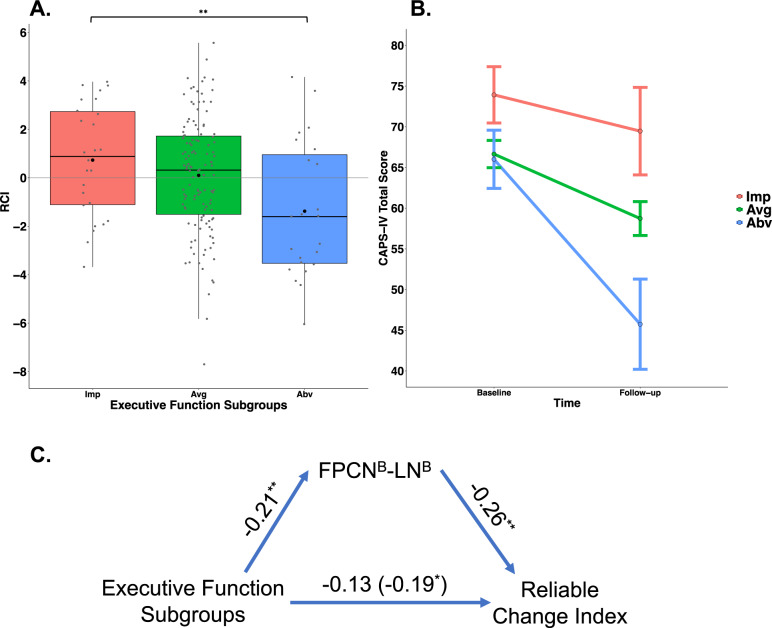
PTSD chronicity. **A** Change in PTSD over time for each EF subgroup. Higher RCI scores indicate increasing scores on the CAPS-IV (worsening symptoms) whereas lower RCI indicates decreasing scores on the CAPS-IV (improving symptoms), ** indicate a significant main effect of EF subgroups predicting reliable change index, *p* = 0.003. Within each box, the dot denotes the mean, and the horizontal line denotes the median. The box indicates the interquartile range (the 25th to the 75th percentile) and vertical line from each box indicates the largest and the smallest value that fall within 1.5 times the interquartile range. **B** Change in PTSD (CAPS) over time for each EF subgroup. PTSD was most chronic in those with impaired EF and improved the most in those with above-average EF. Bars indicate the standard error of the mean. **C** Visualization of the significant mediation model whereby FPCN^B^-LN^B^ functional connectivity mediates the relationship between EF subgroups and change in PTSD over time (RCI). *p *<* 0.05*,* **p *<* 0.01*.* EF executive functioning, RCI reliable change index, Imp impaired EF subgroup, Avg average EF subgroup, Abv above-average EF subgroup, FPCN frontal parietal control network, LN limbic network.

We used linear regression to examine whether PTSD chronicity (i.e., reliable change index; see Methods) differed between the impaired EF-PTSD subtype and the other EF subgroups in those with a PTSD diagnosis at baseline. The EF subgroups significantly predicted PTSD chronicity (*R*^*2*^ = 0.04, *β* = −0.22, *p* = 0.003), such that those with clinically impaired EF at baseline were more likely to have worsening PTSD symptoms, whereas those with above-average EF at baseline showed a decrease in symptom severity (Fig. 3A, B). EF subgroups remained a significant predictor of chronicity after controlling for different categories of covariates including, demographic, clinical, cognitive factors associated with EF, the number of days between baseline and follow-up assessments (*β* = −0.22, *p* = 0.004), or treatment seeking (*β* = −0.34, *p* = 0.017; see Supplemental Results: Follow-up Regression Models and Table S2).

Next, using independent samples *t*-tests, we investigated differences in reliable change index between EF subgroups of PTSD. The impaired EF-PTSD subtype did not differ in chronicity from those with average (*t*(36.74) = 1.23, *p* = 0.215), but did from those with above-average EF (*t*(42.81) = 2.85, *p* = 0.007). In addition, there was reduced chronicity in those with above-average EF compared to average EF (*t*(28.07) = −2.38, *p* = 0.024). These analyses indicate that individuals with PTSD and clinically significant EF impairments had relatively stable and chronic PTSD, whereas those with above-average EF had the most symptom reduction over time (Fig. 3A, B). This suggests a possible additional PTSD-subtype with above-average EF and marked reduction in PTSD symptoms over time.

### PTSD chronicity differences across EF subgroups are mediated by FPCN-LN connectivity

Lastly, we considered how functional connectivity predicted change in PTSD symptoms (i.e., reliable change index) and how it may mediate the EF-chronicity relationship. This analysis was conducted in a sample that had both functional connectivity and longitudinal clinical data (*n* = 121, see Fig. 1). To consider whether FPCN^B^-LN^B^ or FPCN^B^-LN^MT^ functional connectivity improved prediction of PTSD chronicity, we first considered whether functional connectivity between these subnetworks predicted PTSD chronicity. The FPCN^B^s-LN^B^ predicted the reliable change index (*R*^*2*^ = 0.07, *β* = 0.28, *p* = 0.002), while the FPCN^B^-LN^MT^ connectivity did not (*R*^*2*^ = −0.01, *β* = 0.03, *p* = 0.715). Next, we confirmed that EF remained a significant predictor of the reliable change index in this smaller sample (*β* = −0.19, *p* = 0.041). Third, we examined if both FPCN^B^-LN^B^ and EF subgroups predicted unique variance in PTSD chronicity (i.e., reliable change index) when entered as simultaneous predictors in the regression model. In this model (*R*^*2*^ = 0.08, p = 0.002), EF was no longer a significant predictor (*β* = −0.13, *p* = 0.138) while FPCN^B^-LN^B^ remained significant (*β* = 0.26, *p* = 0.005). Finally, we found that FPCN^B^-LN^B^ significantly mediated the relationship between EF subgroups and the reliable change index (Fig. 3C; Average Causal Mediation Effect = −0.238, bootstrap = 1000, 95% CI = [−0.51, −0.02], *p* = 0.026). This analysis suggests that EF may predict PTSD chronicity via its relationship to FPCN^B^-LN^B^ connectivity.

## Discussion

We examined neurobiological and longitudinal evidence for a subtype of PTSD with impaired executive functioning (impaired EF-PTSD subtype). To do this, we examined three normative-based groups with impaired, average, or above-average EF in a sample of post-9/11 veterans with and without PTSD. First, we found that those with impaired EF also had worse memory, attention, PTSD symptoms, and alcohol misuse. Second, we found that the impaired EF-PTSD subtype was characterized by specific frontal parietal control network (FPCN) – limbic network (LN) subnetwork connectivity profiles. Namely, this group exhibited reduced negative connectivity between the FPCN^B^-LN^B^ and FPCN^B^-LN^MT^ subnetworks. Next, we found that this impaired EF-PTSD subtype had more chronic PTSD relative to those with PTSD and above-average EF. In addition, this relationship between EF and chronicity of PTSD was partially mediated by FPCN^B^-LN^B^ connectivity. Critically, these neural and longitudinal associations with the impaired EF-PTSD subtype were robust to accounting for demographics, clinical, and cognitive factors associated with impaired EF. Together, this study provides evidence that an impaired EF-PTSD subtype has a reliable neural signature, and suggests that both EF and FPCN^B^-LN^B^ connectivity impact the longitudinal trajectory of PTSD.

We found that the impaired EF-PTSD subtype had reduced negative connectivity between FPCN^B^-LN^B^ and FPCN^B^-LN^MT^. This is consistent with prominent neurobiological models of PTSD, which suggest that impoverished recruitment of FPCN regions shown to regulate fear, emotion, and mnemonic processing in limbic regions underlies executive and emotional regulation deficits in PTSD [6, 8]. Importantly, this study suggests that this neurobiological mechanism of PTSD is reflected primarily in a subset of individuals with clinically significant EF impairments and only between certain FPCN-LN subnetworks. The FPCN^B^ includes brain regions distributed across the brain including rostral lateral and superior prefrontal cortex, inferior parietal lobule, and middle temporal gyrus [20, 39]. Recent literature indicates that FPCN^B^ is associated with the control of internal mental processes, such as mind wandering [33] and emotion processing [20], and displays increased connectivity with the default mode network [18–20]. Our results indicate that the FPCN has functionally relevant subnetworks that differentially relate to PTSD. The LN^B^ subnetwork—composed of temporal pole regions that are infrequently implicated in the PTSD literature (although see [46, 47]) has shown connectivity to amygdala and hippocampal regions [48] which are more commonly associated with PTSD [5, 49]. These temporal pole regions have been associated with social and emotional processing [21–23] and damage to the temporal pole can lead to unstable mood [21]. The hippocampus and amygdala (LN^MT^) on the other hand are commonly associated with fear, learning, and memory [25]. Our results suggest that dysregulated circuitry underlying the cognitive control of social-emotional processes (FPCN^B^-LN^B^) as well as fear and memory (FPCN^B^-LN^MT^) may characterize PTSD primarily when occurring alongside impaired EF.

The impaired EF-PTSD subtype exhibited reduced negative resting state connectivity, suggesting a reduction in an antagonistic relationship between FPCN and LN subnetworks (i.e., hypoconnectivity). This is consistent with previous intracranial EEG work that identified antagonistic electrophysiological relationships between brain networks [50]. However, the direction of resting state connectivity can be driven by different aspects of pre-processing [51], and describing relative effects across groups is more meaningful. Therefore, an alternative interpretation describes the effect as an increase in functional connectivity between FPCN and LN subnetworks at rest in the impaired EF-PTSD subgroup (i.e., hyperconnectivity). Regardless of the direction of the interpretation (hypoconnectivity vs. hyperconnectivity), the aberrant resting state connectivity in the impaired EF-PTSD group may mark a disruption in emotion or memory regulation [52–54]. Future work using electrophysiological techniques [50] or concurrent TMS and fMRI to identify the causal relationships between networks [55, 56], or task-based imaging to isolate information processing [57], could help adjudicate between these alternative interpretations.

Impaired executive functioning was also associated with worse verbal memory, attention, and other comorbidities such as alcohol abuse. While this is consistent with the transdiagnostic role of EF in risk for a range of psychopathological outcomes [58], the impaired EF-PTSD subtype had unique associations with FPCN-LN connectivity even when accounting for these cognitive and clinical factors. In addition, defining subgroups based on memory or attention did not reveal differences in FPCN-LN connectivity [3]. We also found that those with impaired EF were impaired in an independent inhibitory control task, the gradCPT, which has been previously associated with PTSD symptom severity [35, 36]. Inhibitory control impairments are thought to be the core aspect of EF linked to the development and maintenance of PTSD [7, 35, 59]. For example, both response inhibition and distractor suppression have been uniquely associated with PTSD when compared to other executive functioning measures [35]. Nevertheless, the functional networks and cognitive subgroups of PTSD implicated in this study may be related to other cognitive processes beyond EF. “Cool” EF assessed in this study, such as inhibitory control, are also important for other “hot” emotion-related cognitive processes related to PTSD, such as threat detection, fear learning and extinction, and emotional regulation [5]. As these functions have also been associated with FPCN-LN circuitry [5, 60–62], future work should determine if these EF subgroups are better characterized within the context of these other “hot” cognitive processes related to PTSD.

Executive functioning and FPCN^B^-LN^B^ connectivity predicted differences in the chronicity of PTSD symptoms over a 1–2-year period. The impaired EF-PTSD subtype exhibited more chronic PTSD relative to those with above-average EF, who demonstrated a reduction in symptom severity compared to those with average EF. FPCN^B^-LN^B^ partially mediated the relationship between EF and PTSD chronicity. These results suggest the those with PTSD and above average EF, while they do not exhibit a unique neural signature, are the most distinct with regard to reduced chronicity, and may represent another PTSD-subtype. These results also lend support to the idea that executive dysfunction contributes to the maintenance of PTSD [7, 35] and above-average EF may be a protective factor. Previous work has also identified executive dysfunction as a risk factor for developing PTSD [63, 64] and better EF as indicative of improved treatment outcomes and less dropout [12, 15, 16]. In addition, connectivity in regions associated with emotional regulation and EF predict PTSD symptoms post-trauma [64], consistent with the role of FPCN-LN in mediating the relationship between EF and PTSD chronicity. Together, impaired EF may increase susceptibility to chronic PTSD via reduced negative connectivity between brain networks involved in emotion regulation (FPCN^B^-LN^B^) whereas those with above-average EF have more neurocognitive resources that allows for better recovery after trauma.

One significant limitation of this study is that incomplete data was collected for treatment-seeking between baseline and follow-up. Without detailed information on treatment between baseline and follow-up, we could not with certainty determine whether EF subgroups differ in chronicity due to differences in treatment-seeking behaviors or differences in treatment resistance. However, we observed no differences in baseline medication usage across EF subgroups, suggesting that EF subgroups were not driven by medically-induced impairments at baseline. In a subset of participants, we found that treatment seeking was similar across EF subgroups (86–93%). While treatment seeking individuals were overall more chronic, this effect did not account for the EF subgroup effect on chronicity (Supplemental Results: Treatment Analysis). These results point to the challenge in treating PTSD [65] in that those seeking treatment did not necessarily improve. Although, without treatment type and duration information available, the role of treatment in these EF subgroups remains inconclusive.

Another limitation is that data was not collected immediately post-trauma. Thus, it is unclear if the observed neurocognitive markers are risk factors, caused by chronic PTSD or related to other psychosocial factors such as work, family, and social support. We did observe that estimated pre-morbid verbal abilities differed across EF subgroups and predicted FPCN^B^-LN^B^ connectivity (Table S2). This indicates that FPCN^B^-LN^B^ connectivity may reflect multiple aspects of premorbid cognitive functioning and could be a vulnerability factor for PTSD. Future research that investigates pre-trauma or acute trauma assessments [66] of cognitive ability and brain functioning will help determine how neurocognitive functions serve as risk or protective factors for the development of PTSD and related comorbidities. Additionally, this study has limited generalizability of the impaired EF-PTSD subgroup outside of white male veterans, who may have specific educational background and trauma experiences relative to other less represented individuals. Future work should invest resources towards recruiting a more representative sample to investigate the generalizability of this impaired EF-PTSD subgroup. Finally, future work that uses other neuroimaging approaches such as task-based fMRI may help better identify neurocognitive subtypes of PTSD [67].

Explaining heterogeneity in PTSD is a critical goal to improving treatment and quality of life in victims of trauma. This study examined neurobiological and clinical longitudinal evidence for a cognitive subtype of PTSD. We found that this impaired EF-PTSD subtype exhibited dysconnectivity between specific subnetworks of the FPCN and LN. In addition, these individuals with PTSD and impaired EF had more chronic PTSD after ~2 years. The results suggest treatments personalized for this impaired EF-PTSD subtype should consider targeting EF (e.g., via cognitive training) or these FPCN-LN circuits (e.g., via brain stimulation) to improve PTSD and functional outcomes. Thus, the described impaired EF-PTSD subtype contributes to understanding risk and recovery, personalized treatment approaches, and neurocognitive models of PTSD.

## Supplementary information

Supplemental Methods and Results
