# Supplementary material for: An executive function subtype of PTSD with unique neural markers and clinical trajectories
Source: Transl Psychiatry. 2022 Jun 27;12:262. doi: 10.1038/s41398-022-02011-y (PMC9237057; doi:10.1038/s41398-022-02011-y)
Supplement: Supplementary file 1 — Supplemental Methods and Results [file 41398_2022_2011_MOESM1_ESM.docx]

# Supplemental Methods

## Assessment of PTSD, comorbidities, and demographics.

We considered general demographic factors including age, gender identity, education (years of education), Verbal Ability (total score from the Wechsler Test of Adult Reading [WTAR]^1^), and race. We also considered common comorbidities of PTSD, including: depression and anxiety (depression and and anxiety total scores from the Depression Anxiety Stress Scales [DASS]^2^), mild military TBI (Boston Assessment of TBI-Lifetime [BAT-L]^3^), average alcohol use (average number of drinks on a drinking day from Lifetime Drinking History [LDH]^4^), average pain (average pain in the last month from the McGill Short Form^5^), and sleep quality (global sleep score from the Pittsburgh Sleep Quality Index [PSQI]^6^).

## Performance Validity

Commonly, practitioners and researchers include a measure of effort to help determine the validity of performance on neuropsychological test(s) ^7–9^. As in other studies with this sample^10–14^, we used the Medical Symptom Validity Test (MSVT^15^) to determine validity of performance on neuropsychological tests. The criterion for effort failure on the MSVT as a score of 85% or less on immediate recall, delayed recall, or consistency ^16^. Only those who passed performance validity were included in the initial 368 participants in this study (n = 16 were excluded prior to this initial sample), as the validity of their clinical and neuropsychological data was uncertain^12^.

## Attention and Memory Cognitive Composites

The procedure to calculate cognitive composites has previously been used in a PTSD sample^10–12^. Attention and verbal memory composites were calculated as the average of the age-adjusted z-scores for the measures within each domain. The measures used to calculate the attention and verbal memory composites are reported in Table S1.

## Gradual Onset Continuous Performance Task (gradCPT).

For this study, participants completed either a 4-minute (n = 50) or an 8-minute (n = 57) gradCPT at baseline. The gradCPT is a well validated measure of sustained attention and inhibitory control^17–19^, that has been shown to be sensitive to PTSD and related comorbid conditions^20–22^. In the gradCPT, stimuli consisted of 20 round, grayscale photographs of mountain and city scenes, with 10 from each category. For each trial, a random scene was chosen for presentation with a 90% probability for a city scene and 10% probability for a mountain scene. An identical image could not be chosen for consecutive trials. Scene images gradually faded from one to the next over the course of 800 ms using a linear interpolation, for a total 300 trial images over the 4-minute run or 600 images over the 8-minute run. Note, the 4-min version matched the web-based version previously conducted in over 10,000 participants^19^. Participants were instructed to press a button when a city scene was shown and withhold responses for mountain scenes (i.e., go/no-go task) with emphasis on response accuracy over speed. However, since a new image replaced the previous image every 800 ms, there was an implicit response deadline.

### Reaction time variability and accuracy (d’). Analysis of behavioral performance on the gradCPT has been described in detail elsewhere^17,19^. Task accuracy (d’) and reaction time variability are considered primary measures of the “ability” to sustained attention and inhibitory control. Reaction times to each trial are determined using an iterative algorithm that assigned button presses to individual trials. Reaction times (RT) were calculated relative to the beginning of each image onset. Thus, a RT of 800 ms would indicate that the current image was 100% coherent whereas shorter RT indicates that the current image was still in the process of transitioning from the previous image. After determining RTs, RT variability (defined using the coefficient of variation [CV]) is computed as the standard deviation of all correct RTs divided by the mean of all correct RTs for that participant. To assess accuracy, sensitivity (d’) is calculated using standard signal detection analysis, as the standardized ratio of correct omissions to mountain trials (i.e., “hits”) to incorrect omissions to city trials (i.e., “false alarms”). If participants exhibited 100% or 0% accuracy rates, one-half of an error was respectively added or deducted per standard procedures.

## Neuroimaging methods

### Acquisition and Preprocessing

MRI Acquisition. Two T1-weighted anatomical MPRAGE scans (TR = 2530ms, TE = 3.32ms, flip angle: 7°, 1-mm isotropic) were acquired for inter-participant registration and normalization. Two six-minute T2* weighted fMRI scans (gradient echo-planar imaging – TR: 3000ms, TE: 30ms, flip angle: 90°, 3x3x3.7 mm slices for 38 slices) were acquired during resting state. The same MRI acquisitions were used across both scanners. During rest participants were instructed to keep their eyes open and stay awake.

Image Processing. Resting-state fMRI images were preprocessed using AFNI^23^. This processing pipeline included motion correction, registration to standard space, slice time correction, scan concatenation, censoring of timepoints with a framewise displacement (> 0.5 mm), 6mm FWHM Gaussian smoothing, followed by regression of motion parameters, white matter time series, ventricle time series, global signal, and high-pass filtering via linear, quadratic and cubic detrending. We chose to include global signal regression since it removes motion and respiratory artifacts and previous work suggests that regressing out global signal improves resting-state connectivity/behavior relationships^24^. Control for head motion confounds in resting-state involved removing individuals with greater than 20% of their functional MRI scan censored during preprocessing (n = 12; these 12 were not included in the initial sample size of 368). Those with mean edge-wise functional connectivity greater than 3 standard deviation from the mean were removed (i.e., functional connectivity outliers), and in this sample, no participants met this criteria. The timeseries from each voxel went through additional cleaning steps. First, if the mode of the timeseries value at a given voxel composed more than 20% of the values within that voxel, that voxel was removed due to signal loss. Next, for each timeseries, timepoints censored in preprocessing were imputed via linear interpolation. Finally, in order to reduce the influence of extreme values when computing functional connectivity, outliers in each timeseries that were greater than or less than four standard deviations from the mean were reassigned the threshold value at four standard deviations (i.e., clipping^25^)

*Brain Parcellation.* We used the 200-region 17-network atlas from Schaefer colleagues^26^ which was developed to take advantage of both global and local characteristics of brain organization. Additionally, we included the bilateral amygdala and hippocampus from a subcortical, volumetric atlas developed by Tullo and colleagues^27^. From the 17-network atlas, a total of 4 networks were selected for further study; two limbic networks (LN; LN^A^ and LN^B^) and two frontal parietal control networks (FPCN; FPCN^A^, and FPCN^B^). Additionally, we considered the bilateral amygdala and hippocampus as their own limbic network (LN medial temporal; LN^MT^) bringing the total number of networks that we investigated to five. We explored increasing levels of granularity (300-1000 regions) of this parcellation to determine if the relationship between EF subgroups and FPCN-LN subnetwork connectivity was specific to our original parcellation size. As the parcellation sizes increase, there are slight shifts in parcel and network boundaries. Therefore, if our results are specific to our current parcellation then our results may be spurious or could be specific to certain network boundary parcels. If the results are not specific to our current parcellation, then the results are more generalizable to these networks regardless of local parcel boundaries.

## Reliable Change Index (RCI)

The RCI was calculated using the following formula: $\frac{X}{\sqrt{\left( std\left( T1 CAPS \right)*\sqrt{1-R^{2}} \right)}}$ ^28^. In this formula, *X* represents PTSD symptoms at follow-up (*T2 CAPS*) adjusted for baseline PTSD symptoms ([*T1 CAPS*]; residuals), and *R* is the test-retest reliability of the CAPS-IV^29^ (*R* = 0.89).

## Treatment

To assess how treatment between baseline and follow-up may have contributed to the RCI or differ across executive function subgroups, we were able to cull medical records between baseline and follow-up. From the medical records, we were able to identify those who had sought treatment for PTSD in a subset of 91 participants. Treatment was defined as either medication management, like an SSRI, with at least one prescription refill and/or repeated psychotherapy sessions (excluding AA meetings). These criteria were assessed through clinical notes reported during the clinician administered assessments (e.g., SCID) at follow-up. In addition, medication management was further assessed using participants’ VA medical records dated after the baseline assessment. Importantly, as data were mined from clinical notes/medical records, details were missing in some cases, such as treatment frequency and length of treatment participation. Therefore, this measure should be interpreted with caution. Future work on the relationship between treatment seeking behavior or treatment efficacy in relation to the impaired EF-PTSD subtype should be investigated more rigorously. This current procedure provided a coded variable that identified those who we surmised received treatment (psychotherapy, pharmacological, or both) vs. those who did not.

# Supplemental Results

### gradCPT differences between EF subgroups

This analysis determined the relationship between gradCPT and EF subgroups. The gradCPT was not included in subsequent analyses as this data was only available in a smaller sample of subjects (*n* = 107). EF subgroups significantly predicted both task accuracy (*β* = 0.24, *p* = 0.011) and RT variability (*β* = -0.28, *p* = 0.003). Both accuracy and RT variability effects were robust to controlling for the duration of gradCPT administered (4-minute vs 8-minute, *p* values < 0.01).

## Follow-up regression models

A number of follow-up regression models were conducted with specific categories of covariates in the following manner. Regression models for each outcome variable (FPCN^B^-LN^B^ functional connectivity, FPCN^B^-LN^MT^ functional connectivity, or chronicity/reliable change index) were conducted with the hypothesized predictors of interest (Connectivity models: EF subgroups, PTSD, and EF x PTSD interaction; reliable change index model: EF subgroups). To account for a range of factors related to EF and to examine the specificity of EF as a predictor, three models were repeated for each of the following covariate categories: demographic (gender and verbal abilities [WTAR]), clinical comorbidities (PTSD symptom severity, alcohol use, and sleep dysfunction), and cognitive factors (verbal memory and attention). These covariates were chosen because they differed between EF subgroups in the overall sample. A fourth set of regression models covaried for MRI scanner type and head motion for the functional connectivity outcomes. Finally, for the PTSD chronicity outcome, a fifth set of regression models covaried for days between baseline and follow-up as well as treatment-seeking behavior. Table S2 provides the results from these additional analyses. When including these demographic, clinical, cognitive, and scanner related covariates, only verbal ability significantly predicted FPCN^B^-LN^B^ connectivity (*p* = 0.014), but the interaction between EF and PTSD diagnosis remained significant (*p* = 0.004), see Table S2. None of the demographic, clinical, cognitive factors, nor time between baseline and follow-up predicted PTSD chronicity (*p* > 0.44), with the exception of treatment-seeking between baseline and follow-up. In this model both treatment-seeking (p = 0.03) and EF predicted the Reliable Change Index (RCI; p < 0.001), such that those who sought treatment had more chronic PTSD.

We also found that the EF x PTSD interaction predicting FPCN^B^-LN^B^ and FPCN^B^-LN^MT^ connectivity was consistent across finer parcellations (Table S3).

Further, instead of PTSD diagnosis, we considered if CAPS-IV total symptoms severity or specific symptom clusters (re-experiencing, avoidance/numbing, and hyperarousal) were specifically driving the interaction between PTSD and EF subgroups when predicting functional connectivity (Table S4). Overall symptom severity, and all three symptom clusters interacted with EF subgroups to predict FPCN^B^-LN^B^ and FPCN^B^-LN^MT^ connectivity, indicating that the interactions between PTSD and EF were not specific to any one symptom cluster.

We investigated if the change in scanner influenced the relationship between FPCN^B^-LN^B^ connectivity and RCI. When including both FPCN^B^-LN^B^ connectivity and scanner type as predictors of RCI, only FPCN^B^-LN^B^ remained significant (*R^2^* = 0.06, model *p* = 0.007; FPCN^B^-LN^B^ *β* = 0.28, *p* = 0.002; scanner type *β* = -0.03, *p* = 0.777). Scanner type remained insignificant after adding EF subgroups to this model (*R^2^* = 0.06, model *p* = 0.014; FPCN^B^-LN^B^ *β* = 0.27, *p* = 0.002 scanner type *β* = -0.02, *p* = 0.841; EF subgroups *β* = -0.09, *p* = 0.336). Therefore, scanner type did not influence the relationship between RCI and FPCN^B^~LN^B^ connectivity.

We also considered head motion as a possible confounding predictor of subnetwork connectivity, as the previous literature indicates that head motion covaries with executive function (e.g., Kong et al., 2014^30^). Head motion did correlate with EF subgroups (*r* = -0.12, *p* =0.037) indicating more head motion was associated with impaired EF. Therefore, we conducted linear models to investigate the role head motion played in predicting functional connectivity. After including head motion as a covariate in linear models predicting either FPCN^B^-LN^B^ or FPCN^B^-LN^MT^, head motion nor its interaction with EF was not a significant predictor of functional connectivity (FPCN^B^-LN^B^ : *R^2^* = 0.025, model *p* = 0.026, head motion *β* = -0.20, p = 0.261, head motion by EF *β* = 0.13, *p* = 0.473; FPCN^B^-LN^MT^ : *R^2^* = 0.021, model *p* = 0.040, head motion *β* = -0.22, *p* = 0.221,head motion by EF *β* = 0.20, *p* = 0.283, See Table S2). In all cases, the EF x PTSD interaction remained significant (Table S2). Therefore, although head-motion systematically differed by EF subgroup, this did not impact the interaction between EF-PTSD subgroups predicting FPCN-LN subnetwork connectivity. In order to further determine that the differences in EF were not driven by the “biggest movers” we conducted the same linear models after removing the top six participants with most head motion from the impaired subgroup (*n* = 47 🡪 *n* = 41). After removing the top six “movers”, the correlation between EF and head motion was no longer significant (*r* =-0.02, *p* = 0.760) and the EF x PTSD interaction remained a significant predictor of FPCN-LN subnetwork connectivity (FPCN^B^­- LN^B^: PTSD x EF interaction *β* = -0.54, *p* = 0.016; FPCN^B^-LN^MT^: PTSD x EF interaction *β* =-0.53 , *p* = 0.017). Together, there was no evidence that head motion impacted the functional connectivity results reported in this study.

## Treatment Analysis

Of the 91 participants with available records, 72 (79.12%) reported receiving some treatment between baseline and follow-up (psychotherapy, pharmacotherapy, or both). We conducted an analysis to determine if the differences in treatment seeking and its potential relationship with EF could account for the EF subgroup differences in PTSD chronicity. Between EF subgroups, there was no significant difference in the rate of treatment received (*X^2^* = 3.12, *p* = 0.211). Specifically, 85.71% received treatment in the impaired subgroup, 74.19% received treatment in the average subgroup, and 93.33% received treatment in the above-average subgroup. When predicting chronicity, EF subgroups remained a significant predictor (*p* = 0.015) when controlling for treatment (*p* = 0.045; see Table S2). In this same model treatment-seeking also predicted (*p* = 0.03) predicted RCI, such that those who sought treatment had more chronic PTSD.

Table S1.

| Cognitive Domain | Test | Measure |
| --- | --- | --- |
| Executive Function | Delis-Kaplan Executive Function System (D-KEFS)^31^ | Trails: Number/Letter Switching Scaled Score |
|  |  | Stroop: Inhibition Scaled Score |
|  |  | FAS: Category Switching Accuracy Scaled Score |
|  | Cambridge Neuropsychological Test Automated Battery (CANTAB, [http://www.cantab.com](http://www.cantab.com/)) | Intra/Extra Dimensional Set Shift: Completed Stage Trials Standard Score |
|  | Auditory Consonant Trigrams (ACT)^32^ | Mean 9, 18, 36 second delay number z-score |
| Attention | Test of Variables of Attention (TOVA)^33^ | Response Time |
|  |  | D-prime |
|  | Wechsler Ault Intelligence Scale (WAIS-IV)^34^ | Digit Span Forward Scaled Score |
|  | Delis-Kaplan Executive Function System (D-KEFS)^31^ | Trails: Number Sequencing Scaled Score |
| Memory | California Verbal Learning Test  (CVLT-II)^35^ | Short Delay Free Recall z-score |
|  |  | Long Delay Free Recall z-score |
|  |  | Recognition Hits z-score |

Executive functioning subgroups were defined by the following criterion: scoring less than one standard deviation below the mean on two or more of the tests within the EF domain (impaired subgroup), scoring greater than one standard deviation below the mean on two or more of the tests within each of the domains (above-average subgroup), those meeting neither criterion (average subgroup). Continuous cognitive composites were calculated by taking the average age-corrected z-score of the measures included within each domain.

|  |  | Model Values | | *β* Values | | | |  |
| --- | --- | --- | --- | --- | --- | --- | --- | --- |
|  |  | Adj *R^2^* | *p* | EF | PTSD | EF x PTSD |  |  |
| Original models | FPCN^B^ ~ LN^B^ | 0.02 | 0.020 | 0.07 | 0.54^**^ | -0.59^**^ |  |  |
|  | FPCN^B^ ~ LN^MT^ | 0.02 | 0.020 | 0.08 | 0.58^**^ | -0.55^**^ |  |  |
|  | RCI | 0.04 | 0.003 | -0.22^**^ | - | - |  |  |
|  |  | Adj *R^2^* | *p* | EF | PTSD | EF x PTSD | Gender | WTAR |
| Demographics covariate models | FPCN^B^ ~ LN^B^ | 0.04 | 0.005 | 0.12 | 0.56^**^ | -0.62^**^ | 0.01 | -0.15^*^ |
|  | FPCN^B^ ~ LN^MT^ | 0.03 | 0.023 | 0.12 | 0.60^**^ | -0.59^**^ | 0.07 | -0.07 |
|  | RCI | 0.03 | 0.036 | -0.22^**^ | - | - | 0.01 | 0.02 |
|  |  | Adj *R^2^* | *p* | EF | PTSD | EF x PTSD | LDH | Sleep |
| Clinical covariate models | FPCN^B^ ~ LN^B^ | 0.03 | 0.021 | 0.08 | 0.56^*^ | -0.63^**^ | 0.07 | 0.02 |
|  | FPCN^B^ ~ LN^MT^ | 0.02 | 0.057 | 0.08 | 0.63^**^ | -0.57^*^ | 0.01 | -0.02 |
|  | RCI | 0.04 | 0.029 | -0.22^**^ | - | - | 0.06 | -0.02 |
| Cognitive covariate models |  | Adj *R^2^* | *p* | EF | PTSD | EF x PTSD | Attention | Memory |
|  | FPCN^B^ ~ LN^B^ | 0.03 | 0.020 | 0.10 | 0.50^*^ | -0.56^**^ | -0.08 | -0.05 |
|  | FPCN^B^ ~ LN^MT^ | 0.02 | 0.040 | 0.10 | 0.54^**^ | -0.53^*^ | -0.07 | -0.05 |
|  | RCI | 0.04 | 0.024 | -0.24^**^ | - | - | 0.04 | 0.06 |
|  |  | Adj *R^2^* | *p* | EF | PTSD | EF x PTSD | Scanner | Months^†^ |
| Scanner and Months^†^ covariate models | FPCN^B^ ~ LN^B^ | 0.03 | 0.009 | 0.08 | 0.55^**^ | -0.60^**^ | -0.10 | - |
|  | FPCN^B^ ~ LN^MT^ | 0.03 | 0.011 | 0.08 | 0.59^**^ | -0.56^**^ | -0.10 | - |
|  | RCI | 0.04 | 0.013 | -0.22^**^ | - | - | - | 0.02 |
|  |  | Adj *R^2^* | *p* | EF | PTSD | EF x PTSD | HM | HM x EF |
| Head Motion covariate models | FPCN^B^ ~ LN^B^ | 0.03 | 0.026 | -0.02 | 0.49^*^ | -0.54^*^ | -0.20 | 0.13 |
|  | FPCN^B^ ~ LN^MT^ | 0.02 | 0.040 | -0.01 | 0.56^**^ | -0.54^*^ | -0.22 | 0.20 |
|  |  | Adj *R^2^* | *p* | EF | Treatment |  |  |  |
| Treatment covariate models^a^ | RCI | 0.12 | 0.011 | -0.34^***^ | 0.22^*^ |  |  |  |

Table S2.

Connectivity models were conducted with 314 subjects (see Figure 1) and the RCI (Reliable Change Index) models were conducted with 175 subjects (See Figure 1). ^*^*p* < 0.05, ^**^*p* < 0.001, †Number of months between baseline and follow-up. ^a^Treatment models were conducted on a smaller sample of those that treatment data between baseline and follow-up was available (see Supplementary Methods; *n* = 91). EF = executive function, FPCN = frontal parietal control network, LN = limbic network, MT = medial temporal, Edu = education, Gender = gender identity, WTAR = Wechsler Test of Adult Reading, CAPS = Clinical-Administered PTSD Scale for DSM-IV [CAPS-IV] total score, LDH = average number of drinks on a drinking day from Lifetime Drinking History, Sleep = global sleep score from the Pittsburgh Sleep Quality Index [PSQI], HM = head motion, Attention = attention composite (see Methods), Memory = memory composite (see Methods).

Table S3

| Parcellation | Connectivity | | *β* | *p* |
| --- | --- | --- | --- | --- |
| 300 | FPCN^B^ | LN^B^ | -0.52 | 0.013 |
|  |  | LN^MT^ | -0.51 | 0.016 |
| 400 | FPCN^B^ | LN^B^ | -0.61 | 0.004 |
|  |  | LN^MT^ | -0.67 | 0.001 |
| 500 | FPCN^B^ | LN^B^ | -0.58 | 0.006 |
|  |  | LN^MT^ | -0.58 | 0.006 |
| 600 | FPCN^B^ | LN^B^ | -0.60 | 0.004 |
|  |  | LN^MT^ | -0.64 | 0.003 |
| 800 | FPCN^B^ | LN^B^ | -0.63 | 0.003 |
|  |  | LN^MT^ | -0.57 | 0.007 |
| 1000 | FPCN^B^ | LN^B^ | -0.56 | 0.008 |
|  |  | LN^MT^ | -0.58 | 0.006 |

The interaction between EF subgroups and PTSD diagnosis predicting functional connectivity across 6 additional parcellations provide by Schaefer et al. (2018). FPCN = Frontal Parietal Control Network, LN = Limbic Network, MT = medial temporal, EF = executive function

Table S4

| Symptom Severity |  | Model Values | | *β* Values | | |
| --- | --- | --- | --- | --- | --- | --- |
|  |  | Adj *R^2^* | *p* | EF | Symptoms | EF x Symptoms |
| CAPS total | FPCN^B^ ~ LN^B^ | 0.03 | 0.005 | 0.18 | 0.64^**^ | -0.63^**^ |
|  | FPCN^B^ ~ LN^MT^ | 0.025 | 0.012 | 0.17 | 0.60^**^ | -0.57^**^ |
| B | FPCN^B^ ~ LN^B^ | 0.02 | 0.022 | 0.06 | 0.48^*^ | -0.41 |
|  | FPCN^B^ ~ LN^MT^ | 0.02 | 0.017 | 0.11 | 0.57^**^ | -0.54^*^ |
| C | FPCN^B^ ~ LN^B^ | 0.02 | 0.017 | 0.11 | 0.55^**^ | -0.56^**^ |
|  | FPCN^B^ ~ LN^MT^ | 0.02 | 0.02 | 0.11 | 0.55^**^ | -0.50^*^ |
| D | FPCN^B^ ~ LN^B^ | 0.04 | 0.001 | 0.25^*^ | 0.73^***^ | -0.75^***^ |
|  | FPCN^B^ ~ LN^MT^ | 0.02 | 0.038 | 0.16 | 0.51^*^ | -0.53^*^ |

The interaction between EF and CAPS symptom severity predicting functional connectivity *indicates a *p* value < 0.05, ** indicates a *p* value < 0.01, *** indicates a *p* value < 0.001. CAPS total = Total Symptom severity from the CAPS-IV, B = B symptoms from CAPS-IV (Re-experiencing), C = C symptoms from CAPS-IV (Avoidance/Numbing), D = D symptoms from CAPS-IV (Hyperarousal).

Table S5.

| Model |  |  | Imp vs. Avg | | | Imp vs. Abv | | | Avg vs. Abv | | |
| --- | --- | --- | --- | --- | --- | --- | --- | --- | --- | --- | --- |
|  |  | EF (*β*) | *t* | df | *p* | *t* | df | *p* | *t* | df | *p* |
| FPCN^B^~LN^B^ | PTSD+ | -0.23^**^ | 2.67 | 48.60 | 0.010 | 3.49 | 48.34 | 0.001 | 1.80 | 38.36 | 0.080 |
|  | PTSD- | 0.07 | 0.56 | 29.20 | 0.577 | -0.70 | 41.55 | 0.490 | -1.54 | 44.04 | 0.130 |
| FPCN^B^~LN^MT^ | PTSD+ | -0.21^**^ | 3.92 | 45.46 | 0.0003 | 3.37 | 51.95 | 0.001 | 0.26 | 44.24 | 0.798 |
|  | PTSD- | 0.08 | -0.42 | 25.13 | 0.680 | -0.81 | 39.23 | 0.424 | -0.66 | 39.34 | 0.514 |
| RCI | PTSD+ | -0.22^**^ | 0.71 | 37.27 | 0.481 | 2.53 | 44.27 | 0.015 | -2.45 | 29.30 | 0.021 |

Table reporting post-hoc subgroup comparisons in functional connectivity subnetworks with a significant interaction between PTSD diagnosis x EF subgroups, using 2-sample t-tests (see Figure 2.B-2.C). ^**^*p* < 0.01. FPCN = frontal parietal control network, LN = limbic network, MT = medial temporal, EF = executive function, PTSD- = Individuals without a PTSD diagnosis, PTSD+ = Individuals with a PTSD diagnosis, Imp = impaired EF subgroup, Avg = average EF subgroup, Abv Avg = above-average EF subgroup.

References

1 Venegas J, Clark E. Wechsler Test of Adult Reading. In: Kreutzer JS, DeLuca J, Caplan B (eds). *Encyclopedia of Clinical Neuropsychology*. Springer: New York, 2008.

2 Lovibond PF, Lovibond SH. The structure of negative emotional states: Comparison of the Depression Anxiety Stress Scales (DASS) with the Beck Depression and Anxiety Inventories. *Behaviour Research and Therapy* 1995; **33**: 225–342.

3 Fortier CB, Amick MM, Grande L, McGlynn S, Kenna A, Morra L *et al.* The boston assessment of traumatic brain injury-lifetime (bat-l) semistructured interview: Evidence of research utility and validity. *J Head Trauma Rehabil* 2014. doi:10.1097/HTR.0b013e3182865859.

4 Skinner HA, Sheu W-J. Reliability of alcohol use indices. The Lifetime Drinking History and the MAST. *Journal of studies on alcohol* 1982; **43**: 1157–1170.

5 Melzack R, Katz J. McGill Pain Questionnaire. In: Gebhart GF, Schmidt RF (eds). *Encyclopedia of Pain*. Springer Berlin Heidelberg: Berlin, Heidelberg, 2013, pp 1792–1794.

6 Buysse DJ, Reynolds CF, Monk TH, Berman SR, Kupfer DJ. The Pittsburgh Sleep Quality Index: a new instrument for psychiatric practice and research. *Psychiatry research* 1989; **28**: 193–213.

7 Heilbronner RL, Sweet JJ, Morgan JE, Larrabee GJ, Millis SR. American academy of clinical neuropsychology consensus conference statement on the neuropsychological assessment of effort, response bias, and malingering. Clinical Neuropsychologist. 2009; **23**: 1093–1129.

8 Iverson GL. Outcome from mild traumatic brain injury. Current Opinion in Psychiatry. 2005; **18**: 301–317.

9 Stricker NH, Lippa SM, Green DL, McGlynn SM, Grande LJ, Milberg WP *et al.* Elevated rates of memory impairment in military service-members and veterans with posttraumatic stress disorder. *Journal of Clinical and Experimental Neuropsychology* 2017; **39**: 768–785.

10 Jagger-Rickels A, Stumps A, Rothlein D, Park H, Fortenbaugh F, Zuberer A *et al.* Impaired executive function exacerbates neural markers of posttraumatic stress disorder. *Psychological Medicine* 2021; : 1–14.

11 Esterman M, Stumps A, Jagger-Rickels A, Rothlein D, DeGutis J, Fortenbaugh F *et al.* Evaluating the evidence for a neuroimaging subtype of posttraumatic stress disorder. *Science Translational Medicine* 2020; **12**: eaaz9343.

12 Riley E, Mitko A, Stumps A, Robinson M, Milberg W, McGlinchey R *et al.* Clinically significant cognitive dysfunction in OEF/OIF/OND veterans: Prevalence and clinical associations. *Neuropsychology* 2019; **33**: 534–546.

13 Stumps A, Jagger-Rickels A, Rothlein D, Amick M, Park H, Evans T *et al.* Connectome-based functional connectivity markers of suicide attempt. *Journal of Affective Disorders* 2020. doi:10.1016/j.jad.2020.11.061.

14 Evans T, DeGutis J, Rothlein D, Jagger-Rickels A, Yamashita A, Fortier CB *et al.* Punishment and reward normalize error-related cogntive control in PTSD by modulating salience network activation and connectivity. *Cortex* 2021. doi:10.1016/j.cortex.2021.09.004.

15 Green P. Medical Symptom Validity Test (MSVT) for microsoft windows: User’s manual. 2004.

16 Clark AL, Amick MM, Fortier C, Milberg WP, McGlinchey RE. Poor performance validity predicts clinical characteristics and cognitive test performance of OEF/OIF/OND Veterans in a research setting. *The Clinical neuropsychologist* 2014; **28**: 802–25.

17 Esterman M, Noonan SK, Rosenberg M, Degutis J. In the zone or zoning out? Tracking behavioral and neural fluctuations during sustained attention. *Cerebral Cortex* 2013; **23**: 2712–2723.

18 Fortenbaugh FC, Rothlein D, McGlinchey R, DeGutis J, Esterman M. Tracking behavioral and neural fluctuations during sustained attention: A robust replication and extension. *NeuroImage* 2018; **171**: 148–164.

19 Fortenbaugh FC, Degutis J, Germine L, Wilmer JB, Grosso M, Russo K *et al.* Sustained attention across the life span in a sample of 10,000: Dissociating ability and strategy. *Psychological Science* 2015; **26**: 1497–1510.

20 Dutra SJ, Marx BP, McGlinchey R, DeGutis J, Esterman M. Reward Ameliorates Posttraumatic Stress Disorder-Related Impairment in Sustained Attention. *Chronic Stress* 2018; **2**: 247054701881240.

21 Esterman M, Fortenbaugh FC, Pierce ME, Fonda JR, DeGutis J, Milberg W *et al.* Trauma-related psychiatric and behavioral conditions are uniquely associated with sustained attention dysfunction. *Neuropsychology* 2019; **33**: 711–724.

22 DeGutis J, Esterman M, McCulloch B, Rosenblatt A, Milberg W, McGlinchey R. Posttraumatic Psychological Symptoms are Associated with Reduced Inhibitory Control, not General Executive Dysfunction. *Journal of the International Neuropsychological Society* 2015; **21**: 342–352.

23 Cox R. AFNI: software for analysis and visualization of functional magnetic resonance neuroimages. *Comput Biomed Res* 1996; **29**: 162–173.

24 Li J, Kong R, Liégeois R, Orban C, Tan Y, Sun N *et al.* Global signal regression strengthens association between resting-state functional connectivity and behavior. *NeuroImage* 2019; **196**: 126–141.

25 McNorgan C, Joanisse MF. A connectionist approach to mapping the human connectome permits simulations of neural activity within an artificial brain. *Brain Connectivity* 2014; **4**: 40–52.

26 Schaefer A, Kong R, Gordon EM, Laumann TO, Zuo XN, Holmes AJ *et al.* Local-global parcellation of the human cerebral cortex from intrinsic functional connectivity MRI. *Cerebral Cortex* 2018; **28**: 3095–3114.

27 Tullo S, Devenyi GA, Patel R, Park MTM, Collins DL, Chakravarty MM. Warping an atlas derived from serial histology to 5 high-resolution MRIs. *Scientific Data* 2018; **5**: 1–10.

28 Hinton-Bayre AD. Clarifying Discrepancies in Responsiveness between Reliable Change Indices. *Archives of Clinical Neuropsychology* 2016; **31**: 754–768.

29 Weathers FW, Keane TM, Davidson JRT. Clinician-Administered PTSD Scale: A review of the first ten years of research. *Depression and anxiety* 2001; **13**: 132–156.

30 Kong XZ, Zhen Z, Li X, Lu HH, Wang R, Liu L *et al.* Individual differences in impulsivity predict head motion during magnetic resonance imaging. *PLoS ONE* 2014; **9**. doi:10.1371/journal.pone.0104989.

31 Delis D, Kaplan E, Kramer GL. *D–KEFS Examiner’s and Technical Manual*. Pearson Education: San Antonio, 2001.

32 Stuss DT, Ely P, Hugenholtz H, Richard MT, LaRochelle S, Poirier CA *et al.* Subtle neuropsychological deficits in patients with good recovery after closed head injury. *Neurosurgery* 1985; **17**: 41–47.

33 Henry GK. Probable malingering and performance on the test of variables of attention. *The Clinical neuropsychologist* 2005; **19**: 121–129.

34 Wechesler D. *Wechsler Adult Intelligence Scale (4th ed., WAIS– IV)*. Pearson: San Antonio, 2008.

35 Woods SP, Delis DC, Scott JC, Kramer JH, Holdnack JA. The California Verbal Learning Test (2nd ed.): Test–retest reliability, practice effects, and reliable change indices for the standard and alternate forms. *Archives of Clinical Neuropsychology* 2006; **21**: 413–420.
